# Animal Foetal Models of Obesity and Diabetes – From Laboratory to Clinical Settings

**DOI:** 10.3389/fendo.2022.785674

**Published:** 2022-02-07

**Authors:** Emilia Grzęda, Julia Matuszewska, Kamil Ziarniak, Anna Gertig-Kolasa, Izabela Krzyśko- Pieczka, Bogda Skowrońska, Joanna H. Sliwowska

**Affiliations:** ^1^ Laboratory of Neurobiology, Faculty of Veterinary Medicine and Animal Science, Poznań University of Life Sciences, Poznań, Poland; ^2^ Molecular and Cell Biology Unit, Poznań University of Medical Sciences, Poznań, Poland; ^3^ Department of Paediatric Diabetes and Obesity, Poznań University of Medical Sciences, Poznań, Poland

**Keywords:** obesity, diabetes, brain, sex differences, behaviour, cardiovascular system, prenatal programming

## Abstract

The prenatal period, during which a fully formed newborn capable of surviving outside its mother’s body is built from a single cell, is critical for human development. It is also the time when the foetus is particularly vulnerable to environmental factors, which may modulate the course of its development. Both epidemiological and animal studies have shown that foetal programming of physiological systems may alter the growth and function of organs and lead to pathology in adulthood. Nutrition is a particularly important environmental factor for the pregnant mother as it affects the condition of offspring. Numerous studies have shown that an unbalanced maternal metabolic status (under- or overnutrition) may cause long-lasting physiological and behavioural alterations, resulting in metabolic disorders, such as obesity and type 2 diabetes (T2DM). Various diets are used in laboratory settings in order to induce maternal obesity and metabolic disorders, and to alter the offspring development. The most popular models are: high-fat, high-sugar, high-fat-high-sugar, and cafeteria diets. Maternal undernutrition models are also used, which results in metabolic problems in offspring. Similarly to animal data, human studies have shown the influence of mothers’ diets on the development of children. There is a strong link between the maternal diet and the birth weight, metabolic state, changes in the cardiovascular and central nervous system of the offspring. The mechanisms linking impaired foetal development and adult diseases remain under discussion. Epigenetic mechanisms are believed to play a major role in prenatal programming. Additionally, sexually dimorphic effects on offspring are observed. Therefore, further research on both sexes is necessary.

## Introduction: We Are What Our Mothers Eat

Over the years numerous epidemiological findings and data from animal studies revealed the effects of mother’s nutrition on the development of offspring. Researchers proposed the concept of foetal/early programming, according to which early environmental factors can permanently organise or imprint physiological and behavioural systems. Programming is defined as the process in which environmental factor(s), acting during the sensitive period, affect the structure and functions of tissues and organs, leading to lifelong effects (1, 2). Accordingly, researchers suggested redirecting investigations towards the intrauterine environment rather than the environment in later childhood and indicated that the womb may be more important than home (1).

Although hunger is still common nowadays, especially in Africa, paradoxically, all over the world more people are dying of the health problems caused by overeating rather than malnutrition. This subject is particularly interesting at the time when obesity is a worldwide problem, and it is even greater due to the COVID-19 pandemic. As pandemic-related lockdowns reduced people’s physical activity and increased overeating (the factors contributing to the development of obesity), the rates of these metabolic problems are expected to increase rapidly in our society in the future, also among mothers-to-be and their offspring. It is a well-known fact that the Western pattern diet (WPD), which is common in the US and other developed countries, may chronically activate the innate immune system and inhibit the adaptive immune system. Thus, the WPD impairs adaptive immunity while ramping up innate immunity. This leads to chronic inflammation and severely impairs the host’s defence against viral pathogens, including COVID-19 (3). A report on 4,103 patients with COVID-19 disease in New York City showed that the most important clinical features leading to hospital admission were age >65 years and obesity (4). The author of another study on critically ill adults with COVID-19 admitted to two hospitals in New York City reported that the majority were men aged over 60 years and nearly half of them were obese (5). These findings were also confirmed by the authors of European studies on 165 adult patients with a mean BMI 26, who found that severe forms of COVID-19 were associated with high visceral adiposity (6).

According to the findings of studies on animals and humans, both maternal under and overnutrition/obesity affects the cognitive function and the development of neurological and psychiatric disorders in offspring.

The foetal programming concept suggests that maternal nutritional imbalance (both under- and overnutrition) have a long-term effect on the health of offspring and on the risk of diseases such as diabetes (7). The mechanisms responsible for the effects of maternal undernutrition and obesity on the increased risk of future metabolic disease have not been thoroughly investigated. They include changes in the foetal supply of nutrients, genetic and epigenetic factors. Moreover, as results from animal studies on perinatal high-fat diet (HFD) programming, the potential mechanisms of neural pathways include circulating factors, such as hormones (leptin, insulin), nutrients (fatty acids, triglycerides (TG) and glucose), and inflammatory cytokines.

The aim of this paper was to review the data on maternal undernutrition, obesity, and diabetes provided by the authors of research on humans and animals, and to discuss the potential mechanisms underlying the foetal metabolic programming. The main focus of this review is the foetal metabolic system and central nervous system (CNS), because they seem to be the most vulnerable to the harmful effects of programming (8). The mechanism(s) contributing to the sexual dimorphism of metabolic diseases were also investigated. The detailed analysis of data concerning sexual differences observed in the offspring of malnourished and overnourished mothers is shown in the **Table 1**. The understanding of these mechanisms could provide useful tools for the prevention and treatment of these diseases in offspring.

**Table 1:** Maternal under- and overnutrition induce sex-specific differences in body weight, fat content, metabolic and hormonal status, cardiovascular system and brain and behavioural outcomes in offspring; ↑ - increase; ↓ decrease; - no change.

Outcomes	Species	Maternal undernutrition effects on offspring outcomes	Maternal overnutrition effects on offspring outcomes
BODY WEIGHT	Mice	Food restricted diet (FR/50%) ↓males and females birth weight (9) FR/70% ↓males and females birth weight (10)	High fat diet (HFD) − males birth weight ↓females birth weight (11) High fat high sugar diet (HFHSD) ↑adult males body weight > ↑ adult females body weight (12)
	Rats	FR/70% ↓males and females birth weight (13) Protein restricted diet (PR/9%) ↓adult males body weight (14) PR/9% ↓females birth weight − males birth weight (15) PR/5% ↓adult males and females body weight (16)	HFHSD ↓males and females birth weight ↑adult males body weight > ↑ adult females body weight (17) Cafeteria diet (CAF) − males and females birth weight ↓adult males body weight < ↓ adult females body weight (18) CAF ↑ body weight (adult females) (19)
	Human	Maternal undernutrition ↓ females birth weight > ↓males birth weight (20) ↓ females birth weight < ↓ males birth weight (21) ↓ females birth weight <↓ males birth weight (22)	Maternal gestational diabetes mellitus (GDM) and/or obesity ↑males BMI ↑males - risk of the obesity development in adulthood (23)
FAT CONTENT	Mice	PR/6% ↑ adiposity in males and females which fed HFD (24)	HFD ↑ adult males body fat content ↓ adult females body fat content (25) HFD ↑adult males body fat content > adult females body fat content (26) HFHSD ↑ adult males and females - inguinal fat pad mass (12)
	Rats	PR/10% ↑ adult males deposits of abdominal fat − adult females deposits of abdominal fat ↓ adult males deposits of gonadal fat − adult females deposits of gonadal fat (14) PR/10% ↑ young females body fat content − young males body fat content (27)	CAF ↑adult males perirenal fat mass > ↑ female perirenal fat mass ↑adult males gonadal fat content < ↑ females gonadal fat content (28) CAF ↓adult males fat content < ↓ adult females fat content (18) CAF ↑ abdominal fat in adult males and females (19)
	Sheep	FR ↑ fat mass in adult males − fat mass in adult females (29)	
	Human	Maternal uandernutrition ↓ lean and fat mass in females and males at birth ↑ fat mass in girls > ↑ fat mass in boys (30)	Maternal pre-pregnancy BMI ↑ adult females fat mass (31) ↑ young females fat mass (32)
METABOLIC AND HORMONAL STATUS	Rats	PR/10% ↑ insulin and leptin levels in young males and females ↑ TG levels in young males − TG levels in young females (27)	CAF ↑glucose levels in young females ↑insulin levels in young males < ↑ insulin levels in young females (18) CAF ↑glucose levels in adult females ↑leptin levels in adult females (33) HSD ↑ insulin resistance and oxidative stress in adult males − insulin resistance and oxidative stress in adult females (34) HFD ↑glucose levels in adult females − glucose levels in adult males ↑TG levels in adult females − TG levels in adult males (35) CAF ↑ plasma leptin, insulin and TG levels in adult males and females (19)
	Sheep	FR ↓ POMC expression in adult males − POMC expression in adult females (29)	
	Humans	Maternal undernutrition ↑ LDL level in adults males and females ↓ glucose tolerance in adult males and females ↑ insulin resistance in adult males and females (36)	Maternal obesity ↑ blood insulin concentration at birth in boys < ↑ blood insulin concentration at birth in girls ↑insulin resistance in girls adult life (37) Maternal diabetes ↑insulin resistance in girls adult life (38)
CARDIOVASCULAR SYSTEM	Mice	FR ↑ mean arterial pressure (MAP) in adult males - MAP in adult females (39)	HFHSD ↑ diastolic blood pressure (DBP) in adult males ↑ systolic blood pressure (SBP) in adult males ↑ DBP, SBP in adult females > ↑ DBP, SBP in adult males ↑ Heart rate (HR) in adult males and females (12)
	Rats	PR ↑ risk of hypertension in young males > ↑ risk of hypertension in young females (40) PR ↑ risk of hypertension develops in young males and females (41)	HFD ↑ DBP, SBP in adult females − DBP, SBP in adult males (35)
	Humans	Maternal undernutrition ↑ risk of coronary heart disease in adult females ↑ risk of coronary heart disease in adult males (42) ↑ risk of hypertension in adult females ↑ risk of hypertension in adult males (36)	Maternal obesity ↑ risk of stroke in adult females − risk of stroke in adult males (43) Maternal diabetes ↑ blood pressure during childhood in the males − blood pressure during childhood in the females (44)
BRAIN AND BEHAVIOURAL OUTCOMES	Baboons	FR ↓ working memory in adolescent females − working memory in adolescent males ↑impulsivity in adolescent males − impulsivity in adolescent females (45)	
	Mice	FR/70% ↓ locomotor activity in males and females ↓ recognition memory (NOR) in males and females ↓ synaptophysin level in the female hippocampus (10) PR/8% ↑ anxiety- and depression-like behaviours in males − anxiety- and depression-like behaviours in females (46)	HFD ↓ myelination in the medial cortex in young males − myelination in the medial cortex in young females ↓ memory parameters assessed in the NOR in young males − memory parameters assessed in the NOR in young females (47) HFD ↑ anxiety-like behaviours in adult males − anxiety-like behaviours in adult females (48)
	Rats	LP/9% ↓ locomotor activity in males − locomotor activity in females ↓ feeding behaviour in females − feeding behaviour in males (14)	HFD ↓ memory parameters assessed in the MWM in adolescent males − memory parameters assessed in the MWM in adolescent females (49) HFD ↑ depression-like symptoms in adult males − depression-like symptoms in adult females (50) HFD ↑ anxiety in adult males < ↑anxiety in adult females (51) HFD ↑ anxiety in young females − anxiety in young males ↓ sociability in young females − sociability in young males (52) HFD ↑ anxiety in male and female neonates (53)
	Humans	Maternal undernutrition ↓ total brain volume in boys/men − total brain volume in girls/women ↓ volumes of grey and white matter in boys/men − volumes of grey and white matter in girls/women (54) ↑ ASD, ADHD and schizophrenia in boys > ↑ ASD, ADHD and schizophrenia in girls (55)	Maternal obesity ↓ hippocampal development/volume in boys − hippocampal development/volume in girls (56)

ASD, autism spectrum disorder; ADHD, attention deficit hyperactivity disorder; CAF, cafeteria diet; DBP, diastolic blood pressure; FR, food restricted diet; GDM, gestational diabetes mellitus; HFD, high-fat diet; HR, heart rate; HSHFD, high-fat high sugar diet; LDL, low-density lipoprotein; MAP, mean arterial pressure; MWM, Morris water maze; NOR, novel object recognition; POMC, pro-opiomelanocortin; PR, protein restricted diet; SBP, systolic blood pressure; TG, triglycerides.

## Animal Models of Obesity With Special Emphasis on Diet-Induced Obesity

Studies on different animal species, mostly mice, rats, and sheep, allow us to understand the mechanisms responsible for the development of obesity and diabetes, and search for intervention strategies. Obesity can be induced in animals chemically (with drugs), surgically, genetically, or through diet (57–61). Obesity has multifactorial aetiology and in the light of the increasing occurrence of unhealthy nutrition (e.g. the consumption of fast food), the rising prevalence of obesity suggests that it is caused by environmental factors. The authors of this review focused mostly on diet-induced obesity in rodents and made references to numerous excellent comprehensive reviews of other models of animal obesity (59, 60).

The most popular models of diet-induced obesity are: high-fat (HFD), high-sugar (HSD), high-fat-high-sugar (HFHSD), and cafeteria (CAF) diets. High-fat diets caused metabolic imbalance, decreased energy expenditure and, as a result, increased the body weight of laboratory animals (62, 63). However, there are differences between species and even strains of rodents. Both Wistar and Sprague-Dawley rats can be used as models of HFD-induced obesity (64), although the metabolic effects caused by this type of dietary regimen are more pronounced in Wistar rats. In recent decades the HFD has been most widely applied in experiments on rodents, but there were significant differences in the composition and fat content of the diets provided to animals to induce obesity. The fat in these diets came from multiple sources, including animals (lard, tallow), plants (olive oil, sunflower, corn, coconut), and fish. The fat composition seems to have a major role in obesity because saturated fats cause more deleterious effects than unsaturated fats (63, 65). Another important factor is the concentration of fat in the diet, which usually ranges from 30% to 60%. The diet-feeding period also differed between studies – it usually ranged from 4 to 16 weeks (62, 63).

Similarly to the situation observed in highly developed countries, a high-sugar diet also contributed to the development of obesity in laboratory animals. In experimental models, sucrose was fed separately from the standard feed, as a superadditive or mixed with drinking water (61). The HSD negatively influenced the glycaemic control in rodents. These effects were similar to those observed after feeding an HFD. However, the body weight gain and adiposity in HSD-fed animals were lesser than after feeding an HFD (59).

A combination of HFD and HSD better imitates human characteristics. The HFHSD has widely been used in studies on rodents as a Western pattern diet. However, Omar et al. showed that the HFHSD was not as effective as the HFD in C57BL/6J mice (66).

The CAF diet is employed in laboratory settings, where animals receive a mix of high-fat and high-sugar food products, which are commonly consumed by people (e.g. cake, biscuits, crisps, processed meat, peanut butter, chocolate, cheese, dried fruit) (60, 61, 65). The components of the CAF diet have a high energy value and are highly palatable, which increases animals’ tendency to overconsumption (60, 61, 65). This model mimics the occurrence of obesity in humans as a consequence of a tasty but unbalanced diet. However, this diet varies in the number and type of products used and its energy value [for more details see (67)].

Experimental paradigms may also vary in terms of exposure to these diets, i.e. the length of food consumption before and/or during pregnancy and/or lactation, which may also influence the results. Among the environmental factors influencing the programming of the foetal phenotype, particular attention is paid to disturbed maternal nutrition. Animal and epidemiological studies have indicated that foetal nutritional deprivation is a strong programming stimulus. On the other hand experimental evidence suggests that maternal overnutrition can result in a phenotype of the offspring characteristic of metabolic syndrome. Moreover, both in humans and animal studies, it has been confirmed that prenatal, perinatal and postnatal factors that are associated with disturbed maternal and offspring nutrition are additive (28, 68–71). In consequence, they lead to unfavourable changes in metabolism in adulthood, and induce diabetes. *In utero*, epigenetic changes exacerbate the negative effects associated with the influence of environmental factors throughout life (68–71).

## Animal Models of Diabetes

There are two major types of diabetes, i.e. type 1 (T1DM) and type 2 (T2DM). The latter is the most common, as it represents more than 90% of all cases. T1DM is caused by an autoimmune destruction of the insulin-producing β-cells in the pancreas (72). Due to the pathophysiology of T1DM, insulin therapy is implemented at the onset of this disease. On the other hand, T2DM can be associated with elevated, normal, or low insulin levels, depending on the stage at which the levels of this hormone are measured. This is a progressive disorder, which is manifested by diminishing pancreatic function over time. The authors of this review mostly focused on T2DM occurring during gestation, as it is often associated with obesity and more prevalent than T1DM. Gestational diabetes mellitus **(**GDM) is an issue of particular interest in this review. This is a heterogeneous entity and affects mostly insulin-resistant overweight and obese women. It is also a strong female risk factor for the progression of T2DM (73).

There are multiple animal models used for the induction of diabetes – mostly rodents (mice and rats) as well as sheep, dogs, cats, and other animals. Diabetes can be induced surgically, chemically or genetically. The surgical method requires pancreatectomy, i.e. removal of most of the pancreatic tissue. Diabetes can be induced non-surgically through damage to the pancreatic cells. This effect can be obtained through the administration of drugs such as alloxan and streptozotocin (STZ, toxins destroying β-cells of the pancreas), which cause insulin deficiency and hyperglycaemia in animals. Alloxan diabetogenicity occurs through the rapid uptake of the drug by insulin-secreting cells, the formation of reactive oxygen species, and disturbances in intracellular calcium homeostasis. It is also necessary to remember that the range of the diabetogenic dose of alloxan is quite narrow and even a small overdose may be generally toxic and kill animals due to kidney failure (74).

Like alloxan, the dose range of STZ is narrow. To date researchers have applied single or multiple injections in experimental paradigms with different animal species and strains (75–78). STZ is taken up by pancreatic β-cells *via* the glucose transporter GLUT2, which changes the DNA in these cells and provokes its fragmentation. For more detailed information on the mechanisms of alloxan and STZ action see Szkudelski (74).

A combination of HFD and STZ is often employed in laboratory settings. These two stressors mimic the pathology of T2DM, though on a shorter timescale than the one observed in humans. The use of HFD causes insulin resistance and/or glucose intolerance, while the administration of STZ reduces functional β‐cell mass (79). Another advantage of this model is that it mimics the slow pathogenesis of T2DM occurring in most humans, which progresses from the slow development of an adult-onset diet-induced obesity to glucose intolerance, insulin resistance (and the resulting compensatory insulin release) and, finally, STZ-induced partial β-cell death. Despite its limitations, e.g. the use of two stressors – HFD and STZ, the HFD/STZ is a reasonable animal model of T2DM and represents the late stage of the disease (80).

Sex-dependent differences in the induction of T2DM by STZ are also of particular interest in this review because, according to scientific reports, males are more prone to develop diabetes (81, 82). There are also sex-specific differences in the development and complications of diabetes in humans. For example, diabetes is considered a stronger risk factor for cardiovascular diseases in women than men (81). Women tend to store the fat tissue, whereas men tend to mobilise adipose tissue burning (83). Women exhibit greater insulin sensitivity than men. There are sex-specific differences in body fat distribution, which point to the crucial role of sex hormones (84).

There are also various genetic models of diabetes, but they will not be discussed here because they do not fall within the scope of this review [e.g. see (85)].

Animal models of T2DM provide not only an opportunity to investigate the pathophysiology underlying this disorder, but also enable the assessment of potential strategies for the treatment and prevention of the disease and related complications.

## Animal Models of Undernutrition

To date research on undernutrition has been successfully conducted on various species of animals, such as laboratory rodents (rats, mice), sheep, pigs, and primates. Due to the fact that rodents and sheep are the most commonly used species in this research area, they will be henceforth discussed in this review. For more information on the advantages and disadvantages of other species see the review by Swanson (86).

Perinatal undernutrition can be induced in rodents through either maternal underfeeding (general food restriction or protein content restriction) during gestation or the modification of the offspring’s energy intake during the suckling period. Rodents have been used in numerous studies to examine different degrees of dietary restriction during gestation – from mild (30%), through moderate (50%), up to severe (70%) protein restriction (PR) or food restriction (FR) [for reviews see (87, 88)]. Postnatal manipulations usually include various forms of maternal milk restriction, e.g. maternal deprivation (which promotes perinatal stress) (89–91), early weaning induced with pharmacological compounds such as bromocriptine a drug inhibiting lactation (92)], non-pharmacological early weaning (in the last three days of the suckling period, when nipple suction can be interrupted with a physical barrier, e.g. by wrapping the breast area with a bandage (93–95) or rearing pups in large litters (96, 97). Maternal undernutrition in sheep is usually induced through FR [30-50% of the control feed allowance (98)]. However, there are some changes in the time of nutritional insult.

In conclusion, researchers interested in studying the effects of the maternal diet on offspring in laboratory settings have a variety of options, but due caution is necessary to ensure proper dietary controls and provide a detailed description of the diets used during the experiment, including the time of exposure and considering possible sex differences. The choice of a suitable animal model is a key point affecting the translational potential of the results.

## Effects of Maternal Overnutrition on Body Weight and Fat Content: Animal Studies

**Figure 1:**
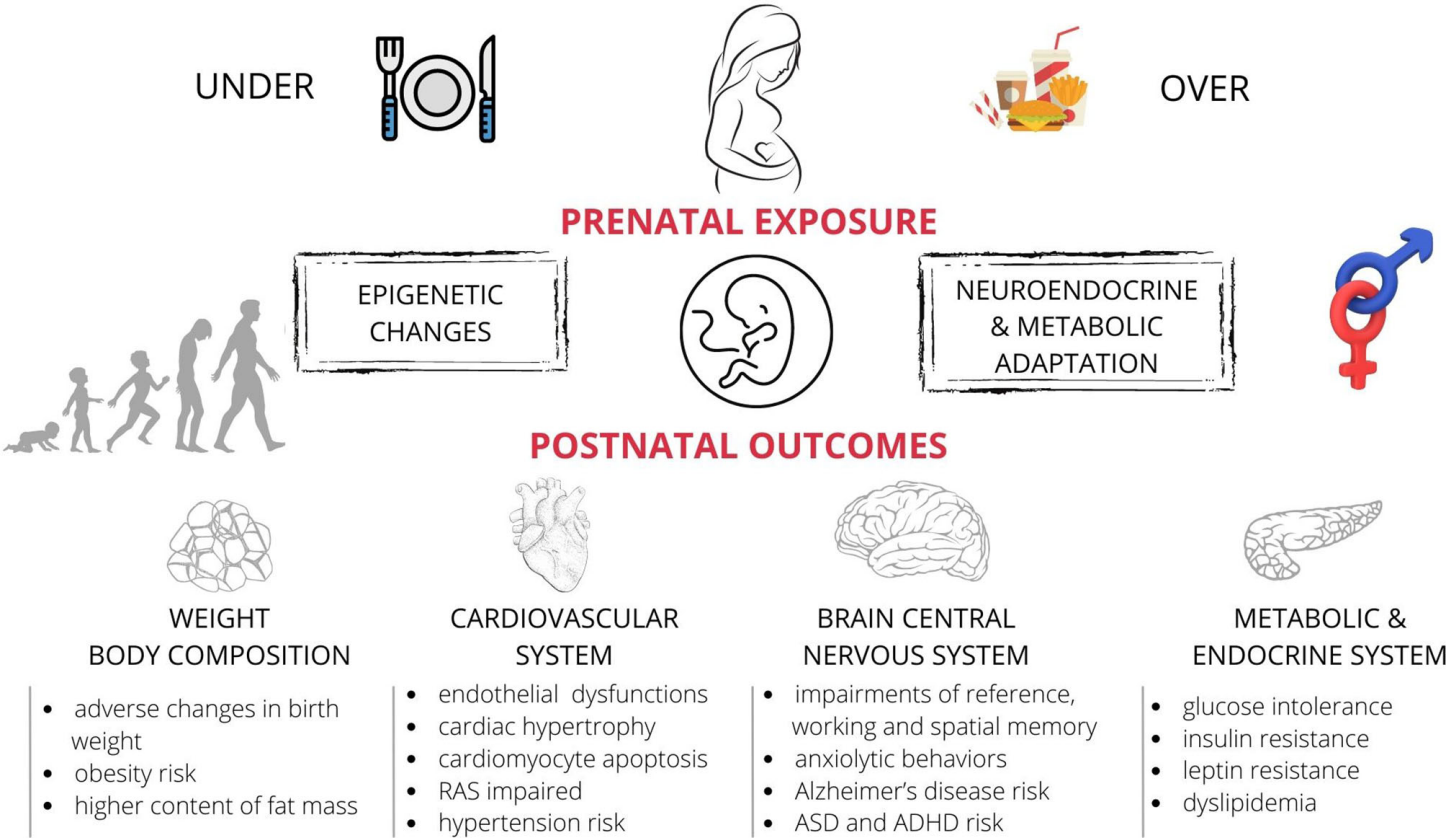
The effects of maternal nutritional imbalance (under- and overnutrition) on offspring during foetal development. Short and long-term negative outcomes (observed both in humans and laboratory animals) include the adverse effects of unbalanced diet on offspring’s metabolism, hormonal state, changes in the body weight and fat content, and abnormal function of the nervous and cardiovascular systems. These effects appear to be sex-specific. ASD, autism spectrum disorder; ADHD, attention deficit hyperactivity disorder; RAS, the renin-angiotensin system.

Maternal overnutrition and/or obesity affect the weight of rodent offspring in a time-dependent manner (**Figure 1**). White et al. conducted a study on rats in which they investigated the relationship between the effects of maternal obesity and an HFD of the offspring on their body weight. They observed that these effects were independent and additive (99). Maternal obesity induced by an HFD caused a significantly lower birth weight of rats (17, 100, 101) and mice (102, 103). Similarly, the offspring of the female rats receiving a CAF diet before and during pregnancy were lighter (28, 104–106). However, other studies showed that the birth weights of the mice delivered by dams exposed to an HFD were significantly greater than those of the control offspring (25, 107). Similarly, the maternal consumption of a CAF diet resulted in a greater body weight of neonatal rats (19). Additionally, several experiments showed that the administration of a CAF diet (70) and an HFD (108, 109) before and during pregnancy did not influence the birth weight of rat and mice pups (110). The differences in the results of various studies may have been caused by the use of different sources of fat and the duration of feeding the diets to the dams and/or offspring (before pregnancy, and/or during pregnancy, and/or lactation), as well as the different effect of obese pregnancies on placental functions. Obesity may increase the placental growth and cause foetal overgrowth or reduce the placental blood flow and limit the foetal growth (111).

Thus, pre-gestational and/or gestational overnutrition of female rodents resulted in a heavier weight of adult offspring, which led to obesity. This effect was observed when: i) the birth weight was significantly lower [rats/HFHSD (17)], ii) the birth weight was not affected (mice/male/HFHCD) (11), and iii) the birth weight was significantly greater [mice/HFD (25, 107)]. Sasson et al. observed that pre-gestational exposure of mice to an HFD restricted the growth of newborn pups, but there was no effect on the weight of adult animals (103). Importantly, studies also revealed a catch-up in the body weight of offspring. The birth weight of the offspring of the female rats fed a CAF diet was either smaller or greater than the weight of the animals in the control group. However, the differences in the body weight disappeared during further development (19, 106).

There is not much data on the sex-specific differences in the effects of maternal diet on the weight of offspring. King et al. found that the maternal HFD did not affect the birth weight of male mice offspring, but reduced the birth weight of females (11). Nivoit et al. found that the offspring of obese rat dams had lower birth weights than the offspring of the animals in the control group. However, when the rats matured, the adult offspring of the HFHSD females became heavier than those in the control group, and these differences were less pronounced in the females (17). Similarly, Samuelsson et al. have found that in the offspring of HFHSD mothers, adult male mice were heavier than females (12). Matuszewska et al. (18) observed that on postnatal day (PND) 25 both male and female offspring of the dams fed a CAF diet had lower body weights than the animals in the control group. However, there was no difference in this parameter on PND 3. Moreover, on PND 25 the CAF females were lighter than the CAF males. On the contrary, in a study conducted by Jacobs et al., feeding mothers the CAF diet resulted in an increase in body weight of adult female rat offspring (19).

As numerous studies on rodents showed, changes in the body weight of the offspring of obese dams seem to be caused by an increase in the body fat content. Increased proportions of the body fat content (112) were described in the rat offspring of females fed HFD (99, 112), HFHSD (17), and CAF diets (18, 28, 70). The authors of the aforementioned studies with the CAF diet observed an increase in the fat content, but there was no change (70) or a decrease in the body weight of the offspring receiving the other two protocols of dietary regime (18, 28). The authors of studies on mice also observed that the fat content in the offspring was influenced by the HFD provided to their mothers (25, 103, 113).

Researchers also observed sex-specific changes in the fat content in offspring. Dahlhoff et al. found that the male offspring of HFD mice had more fat than the animals in the control group, but the female offspring had a lower body fat content than the animals in the control group (25). Masuyama and Hiramatsu (26) observed that maternal obesity induced by an HFD caused an increased fat mass gain in the male and female offspring at 14 weeks of age, but this effect was less pronounced in the females. However, in a mouse model of HFHSD, both adult male and female offspring had increased inguinal fat pad mass (12). Similarly, male and female offspring of rats exposed to CAF diet had higher abdominal fat content (19). Moreover, among the four-week offspring of CAF mothers the males had a higher perirenal fat mass than the females, whereas the females had a greater gonadal fat content than the males (28). Matuszewska et al. (18) observed that both the male and female offspring of mothers receiving a CAF diet for four weeks before pregnancy, and then during pregnancy and lactation, had a lower fat content on PND 25, but these changes were more pronounced in the female CAF offspring.

## Effects of Maternal Overnutrition on Body Weight and Fat Content: Human Studies

Maternal overweight is a risk factor for foetal macrosomia and it increases the risk of development of obesity throughout childhood and adolescence (114–117). High maternal weight during all three trimesters of pregnancy increases the risk of elevated birth weight of offspring (118, 119). The meta-analysis of clinical studies showed that excessive gestational weight gain increased the risk of childhood obesity by 33% (120). The proposed mechanisms underlying these associations include abnormal placental transfer, epigenetic mechanisms, and altered peripheral and central metabolic profile in offspring (114, 115).

A higher pre-pregnancy BMI also increases the total body fat mass during childhood, and abdominal subcutaneous and preperitoneal fat mass (121, 122). Eshrigui et al. (31) indicated a direct linkage between the maternal pre-pregnancy BMI and the fat mass index, the percentage of body fat, the visceral adipose tissue, and the android-to-gynoid fat ratio of offspring.

Cohort clinical studies showed that metabolic disorders resulting from the obesity of offspring were sex-specific (23). The male but not female offspring exposed to gestational diabetes mellitus (GDM) and/or obesity had a higher BMI and were more likely to develop obesity from late childhood to early adulthood (123). Maternal glycaemia appears to be a major factor initiating adiposity in male infants, whereas the maternal BMI is the main predictor in female infants (23). Moreover, Chaparo et al. and Eshriqui et al. found that the maternal pre-pregnancy BMI was directly associated with a greater fat mass in daughters, but not sons (31, 32).

## Effects of Maternal Undernutrition on Body Weight and Fat Content: Animal Studies

Numerous studies assessing the effects of malnutrition on a number of animal models have shown that either food restriction (FR) or protein restriction (PR) can programme offspring to altered adiposity and body weight at different stages of gestation and postnatal life.

In rats both paradigms of maternal malnutrition (regardless of the timeframe of undernutrition, whether energy restriction lasted throughout gestation or only part of this period) often resulted in a lower birth weight (up to ~40%) (124–127). However, most of these studies showed that despite the lower birth weight observed in the offspring, these animals were often characterised by increased adiposity, gain of the adipose tissue or adipocyte size at later stages of life (124, 128, 129). Few studies showed that a lower body weight could persist for several days, weeks (126, 127, 130) or even for up to ten months, especially when the dam’s age was taken into consideration, as Ware et al. (131) observed. In their experiment dams were mated at different ages, i.e. at 2 and 4 months of age (the young maternal group), and at 6 and 9 months of age (the old maternal group). The animals in both age groups were fed a low protein (LP) diet (50% restriction) or a control diet throughout gestation. After weaning both the male and female offspring of these dams were fed a control diet until 9 months of age. After this period the offspring were fed either the control diet or an HFD (40% of fat) for 9 weeks. In general, at 10 months of age the male offspring of the animals fed the LP diet were smaller than the control counterparts. However, the male offspring of the older LP dams had over a 50% lower weight gain than the male offspring of the young LP mothers. The male offspring of the LP females also had less fat and a smaller adipocyte diameter than the animals in the control group, regardless of the dams’ age. On the other hand, although the female offspring exhibited similar changes in adiposity, the maternal diet had no effect on their body weight or weight gain at 10 months of age (131). The offspring of PR mothers also seemed to be more sensitive to the deleterious effects of the HFD as they exhibited apparent catch-up growth to match the body weight of the control counterparts fed the hypercaloric diet (125).

Available data also suggest that the detrimental *in utero* effects of maternal malnutrition can be transmitted transgenerationally, with altered glucose homeostasis (132), number of larger adipocytes (subcutaneous white adipose tissue on the abdomen) in generation F2 (males), as well as an increased area of GFAP-immunoreactive astrocytes, which is a marker of neuroinflammation (124). Importantly, these effects seem to be transmitted mainly through the maternal rather than paternal line (132).

It is also noteworthy that the majority of experiments on maternal malnutrition focused on male offspring. There is also some evidence [e.g. (14, 15, 131, 133)] that exposure to maternal undernutrition programmes the adipose tissue in a sex-dependent manner, as it seems to be the case in humans. Recently Christians et al. published a systematic review and meta-analysis, in which they analysed the effects of PR and FR on the physiological traits of offspring (e.g. body weight, body fat percentage, fat pad weight, concentration of blood lipids), and checked if any of these traits were more severely affected in either sex. In general, the meta-analysis showed that the birth weight of offspring was consistently reduced in both sexes, regardless of the stage of gestation when the PR or FR occurred. The authors also concluded that less than a half of the studies they analysed tested the interaction between the sex of the offspring and the maternal diet, which may have resulted in a higher incidence of false-positive sex-specific effects (87). However, a maternal FR diet in murine (9, 10): and rat (13) models resulted in a decrease in birth weight in both male and female offspring. Zambardo et al. have found that in the rat offspring of PR mothers, females after birth were lighter than males (15). Weight loss has also been reported in the adult rat offspring of PR mothers of both sexes (16), or in males only (14). Bellinger et al. have described that abdominal fat deposits increased only in rat males, but when it comes to gonadal fat – fat content increased only in females (14). Furthermore, Vega et al. have found that body fat content increased in juvenile rat females only (27), while Begum et al. showed that fat content increased in adult sheep males (29).

## Effects of Maternal Undernutrition on Body Weight and Fat Content: Human Studies

World human conflicts and socio-geographical conditions resulted in times of famine and provided unique opportunities to study the influence of undernourishment during pregnancy on the health of offspring in childhood and adulthood (134, 135). Maternal malnutrition during pregnancy results in a small size of offspring at birth, and the low birth weight is interpreted as an indicator of foetal malnutrition (134–136). However, Roseboom et al., who analysed the children of the mothers who were pregnant during the Dutch famine, observed that the babies whose mothers were caught by famine in the first trimester of gestation, were heavier at birth than the population average before and after the period of starvation. On the other hand, the birth weights of the babies affected by the famine in late gestation were usually lower than the birth weights of the babies who were born before or conceived after the famine (137). Additionally, the exposure to the Dutch famine during gestation affected the sex ratio of liveborn babies. The percentage of boys born alive was lower, especially after the exposure to famine during late gestation (137). Interestingly, the offspring of prenatally undernourished fathers, but not mothers during the Dutch famine were heavier and more obese than the offspring of the fathers and mothers who had not been undernourished prenatally (138).

Researchers also observed that malnutrition during the first and second trimesters of pregnancy was associated with an increased prevalence of obesity (regardless of the birth weight) (137), whereas malnutrition in the third trimester correlated with a lower to normal weight in adult life (139, 140). Hence, exposure to famine in early gestation was associated with greater prevalence of obesity in adult life.

The aforementioned human studies showed that maternal overnutrition was associated with macrosomia and increased fat content in progeny, which predisposed them to metabolic imbalance later in life. There were sex-specific differences in these results. On the other hand, studies on humans showed that although the children of malnourished mothers had lower birth weights, they tended to be overweight and obese later in life. This relationship could be explained by the so-called ‘thrifty phenotype’ hypothesis, which proposes epidemiological associations between poor foetal and infant growth, and the subsequent development of T2DM and the metabolic syndrome. This stems from the effects of poor nutrition in early life, which leads to permanent changes in glucose-insulin metabolism (2, 141–143).

The sex-specific birth weight changes in human offspring of children of malnourished mothers have been also reported. Ntenda et al. (21) and Thurstans et al. (22) have found that boys developed greater decrease in body weight than girls after birth, while Sakisaka et al. (20) described the opposite effect where the lower birth weight was more pronounced in girls than in boys compared to the control groups. Andersen et al. have described that along with the decrease in weight in newborns, fat mass decreased in both sexes, however, later in life, fat mass increased and this effect was more pronounced in girls (30).

## Effects of Maternal Overnutrition on Metabolic and Hormonal Status of Offspring: Animal Studies

Maternal overnutrition also affects the metabolic status of animals. The murine and rat offspring of HFD dams had higher blood glucose, TG and/or cholesterol levels (33, 144–147). Moreover, the offspring of obese rat dams fed an HFD, CAF or HSD had higher insulin, leptin, and adiposity levels (145, 146, 148, 149). Research showed that a CAF diet induced obesity more effectively and resulted in a more pronounced increase in the plasma leptin, TG, and cholesterol levels than the HFD protocol (150, 151). Moreover, the maternal HFD, HSD, and CAF diets resulted in a low-grade inflammatory phenotype in the rat offspring. They had higher levels of pro-inflammatory cytokines such as interleukins: IL-1β, IL-6, and tumour necrosis factor α (TNF-α), which mimics the situation observed in obese patients (152–154).

Overnutrition and/or obesity lead to a combination of peripheral and central nervous system alterations and affect the brain circuitry controlling energy homeostasis and adverse programming of central appetite regulators (149). Central leptin and insulin resistance is commonly observed in the offspring of obese HFD-fed rat females (149). Researchers observed changes in the brain appetite regulators, such as downregulated hypothalamic neuropeptide Y (NPY) and upregulated hypothalamic proopiomelanocortin POMC receptor expression in the offspring of HFD-fed rat females (149). Moreover, epigenetic markers at POMC are influenced by both maternal food excess and restriction [for a review see (155)].

Recently researchers have paid more attention to sex-specific differences in the offspring’s response to mother’s overnutrition. For example, there were sex-specific differences in the blood glucose levels of 25-day-old female offspring of dams fed a CAF diet. These animals had higher glucose levels than those in the control group (18). The concentration of insulin was elevated both in the male and female CAF offspring on postnatal day 25 (PND 25), but this rise in insulin levels was more pronounced in the females (18). Similarly, Bayol et al. observed that feeding rats a CAF diet during pregnancy, lactation, and post-weaning period resulted in sex-specific differences in the glucose and insulin levels in the offspring (33). The concentration of glucose in the ten-week-old female offspring of the rats fed a CAF diet was higher than in the control animals. However, in the studies conducted by Bayol and Matuszewska there was no difference in the glucose levels between the male offspring of the CAF rats and the animals in the control group. Khan et al. have shown that glucose and TG levels were raised only in female rat offspring of HFD mothers (35). Another study revealed sex-specific differences in insulin secretion and leptin transcription – the males had a higher circulating insulin level, whereas the females had an elevated leptin transcript level (33). Rodriguez et al. also observed maternal HSD-induced insulin resistance and oxidative stress in the male but not in the female rat offspring (34). Chowen et al. (156) also noted differences between the males and females in their response to obesogenic diets/environments and the possible implication of hypothalamic astrocytes.

Sanchez-Garrido et al. observed that obesity was transmitted to offspring in a sex-specific manner by the paternal line. The male, but not female offspring of rat fathers with HFD-induced obesity had higher body weights and leptin levels. However, these animals did not exhibit glucose intolerance. Moreover, the authors noted a decrease in the luteinising hormone (LH) levels and an exacerbated drop in the testosterone levels in the male offspring. On the other hand, the female offspring exhibited reduced LH response to kisspeptin-10 (157).

## Effects of Maternal Overnutrition on Metabolic and Hormonal Status of Offspring: Human Studies

The children of obese mothers (BMI > 30 kg/m^2^) had higher percentage of body fat, systolic blood pressure, TG, and leptin levels, and developed insulin resistance at the age of 8 (121). An interim analysis of 898 obese and normal weight mothers and their offspring from the Programming of Enhanced Adiposity Risk in Childhood-Early Screening (PEACHES) (158) cohort study showed that the children of obese, GDM-negative mothers with late-pregnancy dysglycaemia had a worse outcome with higher weight gain (150–154) and a higher BMI in early childhood than the children of obese mothers treated for GDM (158). Furthermore, the Helsinki birth cohort study showed not only a positive correlation between the BMI of the mothers and offspring, but also a higher body fat percentage in the children of the mothers with a higher BMI (159). The study also showed that the higher maternal BMI increased the risk of the offspring’s death, cancer, stroke, coronary heart disease, and T2DM, with the latter two being the most strongly correlated. Apart from that, the risk of developing TDM2 was higher in women than men, while the incidence of coronary heart disease was greater in men (43).

Moreover, exposure to endocrine disruptors (EDs), which becomes increasingly widespread in the environment, influences weight regulation and has obesogenic effects on humans (160–163). EDs act as hormones at low but persistent doses and mostly mimic oestrogen properties. They activate or inactivate cellular receptors, cell responses, and other targets, and lead to higher insulin resistance and hyperinsulinaemia. EDs also change the adipokine level in a sex-specific way. For example, in the Canadian Maternal-Infant Research on Environmental Chemicals Study, newborns exhibited sex-specific differences in the leptin and adiponectin levels, caused by the maternal exposure to bisphenol A (BPA) *in utero*. The female offspring had higher leptin levels than males. By contrast, adiponectin did not differ between the sexes, but was inversely related to the BPA level in the males (164). The topic of environmental chemicals in the context of parental/early exposure to them and programming of obesity and diabetes type 2 is of great importance, but beyond the scope of this review. For reviews on this subject, see e.g. (165–167). Moreover, the concept of transgenerational inheritance – an endocrine of an adverse outcome after a chemical exposure was proposed in 2005 and discussed later by other researchers [e.g. (165, 168, 169)].

The risk factors leading to the development of T2DM also include sugar-sweetened beverages (SSBs). The meta-analysis of prospective cohort studies showed that the men and women drinking SSBs in the highest quantile had a 26% excess risk of developing T2DM than those in the lowest quantile (170).

Male and female offspring seem to adapt differently to the milieu created by maternal obesity and diabetes during pregnancy (171), which may be connected to the sex hormone regulation of several genes involved in both the physiological control of metabolism and the pathophysiological background of cardiometabolic diseases (172, 173). Shields et al. (37) and Krishnaveni et al. (38, 174) noted that girls not only had a higher cord blood insulin concentration at birth than their male peers, but when exposed to GDM *in utero*, they were more likely to develop insulin resistance later in life. Studies also suggest that androgen excess during pregnancy may play a crucial role in insulin resistance programming in female offspring (175). The teenage daughters of mothers with polycystic ovarian syndrome (PCOS) or congenital adrenal hyperplasia (CAH) had hyperinsulinaemia (176, 177).

## Effects of Maternal Undernutrition on Metabolic and Hormonal Status of Offspring: Animal Studies

The offspring of undernourished rat dams tend to exhibit numerous metabolic and hormonal changes in the postnatal period. There are relatively frequent alterations in insulin signalling and glucose tolerance, transcriptional and protein changes in the adipose tissue, liver, and pancreas, as well as inflammatory markers. Changes in insulin signalling and glucose tolerance involve hyperglycaemia (either after fasting or in non-fasted animals), lower insulin sensitivity, as well as higher insulin increment during glucose tolerance test (GTT) (126, 128, 178). This metabolic imbalance can be accompanied by increased blood concentrations of TG and low-density lipoproteins (LDL), as well as decreased concentration of high-density lipoproteins (HDL) (128, 130). Moreover, there was a strong positive correlation between the plasma TG concentration and the body weight of the offspring of the dams fed a diet with 70% FR throughout the gestation (130).

According to data, the livers of the offspring of malnourished mothers are also highly affected, but the results vary across studies. On the one hand, the male offspring of rat dams subjected to severe PR in a diet (80% restriction in the diet protein content) during the second half of gestation exhibited hepatic steatosis at the age of 90 days (128), whereas Lecoutre et al. observed that 70% FR during gestation and lactation did not affect the liver mass or the content of liver lipids (also in male offspring, aged 4 months) (179). This observation is consistent with the results of the study conducted by Morris et al. on the male offspring (aged 110 days) of FR dams (70% FR throughout gestation), whose liver weight did not change, either. Interestingly, a lower liver weight was observed in a group of younger rats (aged 55 days). Transcriptional liver profiling in the group of younger animals revealed that although these individuals did not yet exhibit the characteristics of the metabolic syndrome phenotype that was observed in older rats, the results suggest that these animals might exhibit metabolic abnormalities in advance of the full metabolic syndrome phenotype observed later in life (i.e. at 110 days of age) (180). The effects of maternal malnutrition during pregnancy on the liver may also be transgenerational and transmitted mainly through the maternal line, as described by Hanafi et al. The F2 foetuses of F1 females fed an LP diet for 2 months after weaning (60% PR) exhibited an overexpression of GLUT2 and glucokinase (GK) in the liver. Moreover, these genes as well as UCP2 were also overexpressed in the pancreas, which suggests that these F2 offspring might be predisposed to diabetes later in life. Indeed, higher postnatal levels of fasting blood glucose, insulin, and HOMA-IR later in life (at the age of 20 and 30 weeks) indicated that maternal undernutrition significantly altered glucose tolerance in generation F2 (132).

Research has shown that maternal malnutrition highly affects the adipose tissue function. Guan et al. observed changes in 650 genes in the visceral adipose tissue in the male offspring of dams subjected to 60% PR throughout pregnancy and lactation. The analysis of the results revealed a global upregulation of the genes involved in carbohydrate, lipid, and protein metabolism, as well as adipocyte differentiation and angiogenesis. This suggests that maternal PR during gestation and lactation programmes the susceptibility to visceral adiposity later in life (181). Importantly, research findings also indicate that the restriction of the mother’s calorie intake (20% restriction, gestational days 1-12) significantly affects the function of the brown adipose tissue (BAT) in the offspring of both sexes (aged 25 days) (182). Maternal malnutrition may also induce epigenetic changes in the adipose tissue of the offspring, e.g. in the means of programming changes in the miRNA or CpG site methylation [for a review see (179)]. Sex-specific alterations (e.g. in the POMC expression) were observed in the offspring of food-restricted sheep. Begum et al. noted a lower POMC expression (accompanied by increased fat mass in adulthood) in the male adult sheep which had been undernourished during early pregnancy, but there was no difference in the females (29). In rats, offspring of PR mothers (both juvenile males and females) had elevated insulin and leptin levels, but TG levels were elevated only in males.

## Effects of Maternal Undernutrition on Metabolic and Hormonal Status of Offspring: Human Studies

The spectrum of maternal malnutrition spans undernutrition with caloric and/or macro- and micronutrient restrictions during pregnancy. Research has shown that the timing of nutritional restriction seems to play an important role on the health of offspring later in life. Studies on the offspring of the mothers who were pregnant during the Dutch famine (183), Biafran (184), and Chinese (185) provided data on the consequences of maternal caloric restriction in offspring, who developed glucose intolerance, microalbuminuria, an atherogenic lipid profile with a higher LDL/HDL ratio, and hypercholesterolaemia (186). Roseboom et al. indicated that the exposure to the famine at an early gestational age was correlated with an increased risk of coronary heart disease, atherogenic lipid profiles, and higher adiposity (187). Painter et al. (188)observed that the exposure to the famine in the middle of gestation was related to microalbuminuria and impaired renal function. The children whose mothers were malnourished during pregnancy had lower insulin levels and insulin-like growth factor 1 (IGF-1) concentrations, but higher proteolytic activity of the insulin-like growth factor binding protein (the protein binding and regulating the IGF activity) immediately after birth. This may have affected the growth of the foetus (189–191). The proper nutrition of children after birth causes a rapid increase in the insulin and IGF-1 levels, which may result in a rapid weight gain, insulin resistance, and T2DM in adulthood (referred to a as the catch-up growth hypothesis) (189, 191).

Researchers found a correlation between the low birth weight and increased risk of developing overweight and obesity, altered body composition, T2DM, hypertension, and cardiovascular disease (24). This evidence is in line with the data from the aforementioned epidemiological studies on individuals exposed to the Dutch famine *in utero*. There was a correlation between the exposure to famine *in utero* and the development of diabetes (192), high blood pressure (193), and impaired body composition of the offspring later in life (24, 139).

Similar data were presented by Finer et al., who researched the metabolic outcomes of exposure to famine during developmental life in rural Bangladesh. Their research additionally showed that gestational and postnatal windows of exposure had variable effects on the offspring’s phenotype (194).

These data could be explained by the thrifty phenotype hypothesis, first described by Hales and Baker. According to the hypothesis, suboptimal nutrition *in utero* may result not only in impaired growth of the foetus and children being born small for gestational age/with intrauterine growth retardation (SGA/IUGR), but also in the reprogramming of metabolic pathways to ensure foetal survival in the challenging *in utero* milieu. However, when these individuals experience the postnatal environment with excessive or normal nutrition, they are more prone to develop impaired glucose tolerance (2, 141–143).

In summary, similarly to the animal data, the studies on humans showed marked alterations in the metabolic and hormonal profiles of the offspring of obese, diabetic, and undernourished mothers. There are emerging reports suggesting that female offspring (animal studies on overnutrition) and girls (human studies; exposure to GDM *in utero*) may be more vulnerable to insulin resistance later in life (36). However, further research on sex-specific differences needs to be conducted, especially to reveal the underlying mechanisms. Studies also have shown that the timing of nutritional restriction seems to play an important role on the health of offspring later in life.

## Effects of Maternal Overnutrition on Offspring Cardiovascular System: Animal Studies

Animal studies, mainly on rodents (rats and mice) (12, 195), but also on sheep (196–198) and non-human primates showed that maternal overnutrition affects the cardiovascular system (199) (**Figure 1**). The maternal HFD caused cardiac hypertrophy and reduced the vascular density of the cardiac muscle in rodents (200, 201) and primates (199). Moreover, lactational overnutrition (resulting from the reduced litter size) altered the cardiac gene expression in rodents. This may impair cardiac anatomy/metabolism and increase the susceptibility to myocardial injury after an ischaemic insult (202). The potential mechanisms related to myocardial dysfunction include impaired cardiac insulin signalling, metabolic status, increased oxidative stress, inflammation, and mitochondrial dysfunction (200, 201). In the sheep model of maternal obesity, in which the offspring’s entire heart function was evaluated in the Langendorff model (an *in vitro* system), the contractile function of the heart was impaired (198). Moreover, the phosphorylation of AMP-activated protein kinase (a cardioprotective signalling pathway) was reduced in the hearts of the foetuses of obese mothers, but the stress-signalling pathway p38 MAPK was upregulated. This indicated impaired cardiac insulin signalling, as compared with the animals in the control group (198). In animal models (especially mice and rats) maternal obesity altered DNA methylation, which was responsible for the abnormal fat metabolism (203, 204), and histone acetylation in the promoter regions of adiponectin and leptin (107). These changes had the atherogenic effect because they increased the adhesion of particles circulating in the blood vessels. In consequence of these epigenetic changes the levels of LDL and free fatty acids increased and altered the nitric oxide synthase (NOS) function, which is a well-known factor in the development of hypertension (205). Maternal overnutrition may also alter specific gene expression and result in cardiomyocyte hypertrophy and abnormal heart development (206, 207).

The exposure of developing mouse and rat offspring to maternal obesity led to their hypertension in adulthood, which deteriorated with age (12, 208, 209). Maternal obesity also programmes the vascular system in offspring. Endothelial dysfunction was observed in the small mesenteric arteries of all offspring whose mothers consumed a fat-rich diet. This showed that this disorder commonly occurs with elevated blood pressure. Endothelial dysfunction may be a consequence of insulin resistance and could reflect the activation of inflammatory pathways as a result of increased adiposity (210).

Sex-specific differences were observed in the offspring of obese mice and rats. Both male and female offspring had elevated blood pressure, but it was more pronounced in the females (12, 35). These studies suggested that female offspring exhibited greater sensitivity to overnutritional insults during foetal life and the cardiovascular development. The following factors influence the development of hypertension in offspring: increased sympathetic activity, increased renal norepinephrine concentration and renin expression, as well as vascular conditions (12, 209, 211).

## Effects of Maternal Overnutrition on Offspring Cardiovascular System: Human Studies

Multiple observational studies on humans revealed a correlation between maternal obesity before and during pregnancy and an increased risk of cardiovascular anomalies (114, 115, 212, 213). The exposure of the foetus to maternal obesity increased its blood pressure, altered the vascular system (endothelial function), and myocardial function (199, 214). Researchers observed a positive correlation between maternal obesity and/or gestational weight gain and higher systolic blood pressure in the offspring (114, 215–217). Moreover, a cohort study showed that a higher maternal BMI increased the risk of hospital admissions of adult offspring due to cardiovascular events (43, 213). Additionally, the vascular markers of endothelial dysfunction were increased in the children exposed to maternal diabetes and obesity during foetal life (218). Atherosclerosis may also be influenced by the maternal diet and/or maternal hypercholesterolaemia, which may cause the development of this disease in offspring (219). The inherent risk of hypertension and vascular dysfunction resulting from maternal obesity, cardiac hypertrophy, and contractile dysfunction is also observed in the offspring (200). Epidemiological studies on humans showed that epigenetic mechanisms were involved in the cardiological consequences of maternal obesity and/or diabetes (206). Moreover, the epigenetic mechanisms of altered metabolic control in the offspring of obese mothers may cause abnormal placental function, altered cholesterol circulation, and higher blood pressure in humans (206, 220, 221).

Only a few studies described the influence of sex on the programming of cardiovascular risk, following a pregnancy complicated by obesity and/or diabetes. Ericsson et al. noted a correlation between maternal obesity and stroke only in the female offspring (43). Aceti et al. conducted a systematic review and observed a strong correlation between maternal diabetes and increased blood pressure in the offspring during childhood, with a stronger correlation in the male rather than female offspring (44).

## Effects of Maternal Undernutrition on Offspring Cardiovascular System: Animal Studies

Hypertension (40, 196, 222, 223), coronary heart disease/heart hypertrophy (40, 224), and vascular dysfunction (225) are among the disorders observed in the offspring of undernourished females in experimental animal models. A global restriction of maternal food intake (30-70% food restriction) during pregnancy resulted in arterial hypertension both in murine (222) and rat offspring (40, 226). There were similar observations made on sheep (196) and cows (223). Studies on pregnant rats (227, 228), mice (229), and sheep (230) showed that a reduced maternal dietary protein intake programmed hypertension in offspring. The elevated arterial blood pressure observed in these animals may have been caused by the increased cardiovascular sympathetic tone (227), suppressed activity of the hypothalamic-pituitary adrenal (HPA) axis, impaired function of the renin-angiotensin system (RAS) (231), and/or altered structure and function of the vessels and the cardiac muscle (40, 224). Torrens et al. and Ozaki et al. observed that the dietary protein restriction or global undernutrition in rats compromised the maternal cardiovascular adaptations to pregnancy and led to the endothelial and peripheral artery dysfunction in the offspring (40, 224). The sheep offspring exposed to a maternal LP diet throughout the foetal period developed right and left ventricular hypertrophy (232). Cardiac enlargement was also observed in the offspring of the rats fed an LP diet (233). In addition, they had a lower heart weight, which was associated with an increased rate of cardiomyocyte apoptosis and a lower total number of cardiomyocytes per heart at birth (234). Maternal undernutrition also affects the epigenetic mechanisms that control and change the transcription of genes involved in the cardiovascular homeostasis. A study on rats showed that the offspring of the females which received a LP diet had incorrect histone modifications of a specific enzyme, and these changes were associated with an increase in the blood cholesterol level (235). A study on mice showed that the offspring of the females which received a restricted diet had cardiovascular diseases in adulthood (236). Studies on animals showed that a restrictive diet of rat mothers during pregnancy resulted in significant miRNA dysregulation in the offspring’s heart (237). Specific miRNA molecules responsible for maintaining the elasticity of blood vessels were inhibited (238), which reduced vascular contractility. Altered global DNA methylation was observed in the offspring of rodent females receiving a restrictive diet. It caused the dysregulation of the renin-angiotensin system in rats (239) and endothelium-dependent artery vasodilation in mice (225). Few of these epigenetic-dependent changes can induce the development of arterial hypertension in offspring. Barros et al. hypothesised that epigenetic mechanisms were involved in the overactivation of the sympathetic nervous system in the offspring of mothers fed an LP diet (227).

The authors of studies on the functioning of the circulatory system in the offspring of malnourished mothers did not usually describe sex-specific differences. However, Ozaki et al. found that hypertension developed more rapidly and severely in the male rather than female rat offspring exposed to a maternal LP diet during pregnancy (40). Additionally, male, but not the female mice offspring of malnourished mothers had elevated mean arterial pressure (MAP) (39). Elmes et al. observed that the effects of the mother’s LP diet on the increased blood pressure of rat offspring were not sex-specific (41).

## Effects of Maternal Undernutrition on Offspring Cardiovascular System: Human Studies

For ethical reasons there is not much data on the influence of maternal malnutrition on cardiac disorders in their children. However, scientists try to analyse the effects of famine in different areas of the world. The analysis of data coming from the offspring of the mothers who starved during pregnancy (e.g. during the Dutch Hunger Winter and during the Second World War) indicate that exposure to famine during foetal life increases the risk of cardiovascular diseases (219, 240). Research has also shown that the people who were exposed to malnutrition *in utero* more often develop coronary heart disease and hypertension (241–243). According to the results of Le Clair et al. (42) and Barker (36), the risk of developing hypertension and coronary heart disease increases in both sexes. Maternal FR causes changes in miRNA, which alters the level of vascular endothelium-derived growth factor involved in the proper maturation and differentiation of the endothelium and its receptors (244).

To sum up, the data presented in the aforementioned animal studies on rodents, sheep, and non-human primates as well as the observations conducted on humans showing the effects of diet on the cardiovascular system clearly indicate that the maternal diet has influence on the cardiovascular system of offspring. Various mechanism responsible for these alterations have been proposed, with a special focus on epigenetic mechanisms. So far the sparse data from animal studies have suggested that female offspring exhibit greater sensitivity to overnutritional insults affecting the development of the cardiovascular system during foetal life. However, it is necessary to conduct further research, especially on animal models of undernutrition as well as the children of undernourished mothers, to analyse sex-specific differences.

## Effects of Maternal Overnutrition on Brain and Behavioural Outcomes in Offspring: Animal Studies

Animal models of maternal overnutrition have provided evidence of persistent changes in offspring’s cognition and behaviour, and complemented human epidemiological data with potential insights into the mechanism linking maternal excessive food intake to adverse neurodevelopmental and psychiatric outcomes in offspring (**Figure 1**). Studies across different animal species indicated effects of maternal overnutrition on memory impairment e.g. working and spatial memory (mice 240, and rats 91, 241, 242).

The study by Robb et al. (49) confirmed a sexually dimorphic effect in the Morris water maze performance, where males performed worse than females. Graf et al. observed that the mice pups whose mothers consumed an HFD exhibited altered myelination and neurobehavioural deficits, and these effects were sex-dependent (47). The disturbed myelination in the medial cortex was observed in the male but not female offspring of HFD-fed dams. These structural changes were correlated with changes in the males’ behaviour only (assigned in the novel object recognition test).

Research has also shown the relationship between maternal obesity and depression. Young adult male rodents exposed to an HFD either *in utero* (48) or during lactation (50) exhibited higher rates of depression-like symptoms than the offspring of control group. The rodent offspring of the dams fed an HFD before mating and during gestation and lactation exhibited more anxiolytic behaviours than the control offspring (245, 246). The authors of some studies on animals also suggested that maternal obesity may increase the risk of neurodegenerative diseases such as Alzheimer’s disease in offspring (246, 247).

The following mechanisms might be responsible for behavioural alterations in the offspring of dams fed a CAF diet and HFD: reduced expression of neurotrophins, lower concentrations of synaptophysin and BDNF, which are involved in the development of memory (248, 249). Studies on rodents showed that maternal overnutrition hindered the neuronal growth and maturation by altering differentiation, neurogenesis, and disruption in apoptotic processes in such areas of the brain as the hippocampus, hypothalamus, and the cerebral cortex (102, 250, 251). Apart from the impaired neuronal anatomy and function, the offspring of HFD-fed dams exhibit altered neurotransmission (252). Research on rodents showed that the serotonin axon density and embryonic neuronal survival in the brain regions critical for behavioural regulation were reduced (250, 253). The offspring of female mice and rats fed an HFD had impaired mesolimbic dopaminergic signalling, which was associated with impaired reward response to food (254, 255). The levels of expression of the N-methyl-D-aspartate receptor (NMDA) receptor subunit NR2B in the hippocampus of rat offspring were significantly reduced in response to maternal overnutrition (249). The adult offspring of mice exposed to maternal obesity and an HFD during gestation and lactation were also characterised by increased hippocampal expression of GABA_A_ receptor (256). Increased anxiety in the rat offspring resulted in higher glutamatergic activity in the prefrontal cortex. It was associated with reduced inhibitory input into the glutamate synapses through the cannabinoid/GABA receptors, whose expression decreased. These changes were sex-dependent, and they were more intense in the males than females (51). On the other hand, Kang et al. found that maternal HFD increased anxiety and decreased the sociability of the female offspring only (52). The study on non-human primates (Japanese macaques) showed that the maternal HFD caused behavioural changes in the female offspring, which exhibited higher anxiety. Instead, male exhibited increased aggression (257). On the other hand, the observed increase in the anxiety-like behaviour was not sex-specific in the murine (256) and rat (53) offspring of mothers fed an HFD.

Researchers have suggested multiple mechanisms underlying the effects of maternal HFD on the offspring’s brain, e.g. increased hippocampal lipid peroxidation, microglial activation in pups, and increased peripheral and central proinflammatory cytokine expression in the post-weaning and adult life (245). Other suggested mechanisms include: dysregulation of insulin, glucose, and leptin signalling in the developing brain (notably in the regions involved in behavioural regulation such as cortex, amygdala, thalamus, hippocampus and hypothalamus) (250, 258). Animal studies have also corroborated associations between maternal obesity, placental inflammation, foetal brain inflammation, and abnormal neurodevelopment in offspring (259). Moreover, these multidirectional changes associated with long-term effects of poor maternal diet are often accompanied by epigenetic changes [for details see the review by Moody et al. (252)]. It is important to note that there have been few studies on sex-specific differences in early programming of behaviour and brain function (as compared with data on males alone) and further research is necessary.

## Effects of Maternal Overnutrition on Brain and Behavioural Outcomes in Offspring: Human Studies

According to the findings of recent studies on humans, maternal overnutrition/obesity affects the cognitive function and the development of neurological and psychiatric disorders in offspring (55). Research has provided evidence that there is a correlation between maternal overnutrition and/or obesity and the poorer cognitive performance of offspring, including lower IQ, poorer motor, spatial, and verbal skills (246, 260, 261). Maternal overweight is a predictor of children’s poorer psychosocial development, as evidenced by lower social competence and increased risk of depression and anxiety (262–264). Brain imaging techniques are very valuable in this respect, but they are still relatively underrepresented. An MRI study revealed a significant correlation between prenatal exposure to maternal obesity and a smaller hippocampal volume in boys but not girls (56). Moreover, sex-dependent differences were observed across hippocampal subfields (CA1, CA2/3, CA4, dentate gyrus, and subiculum) (56). Thus, the study suggested that boys may be more vulnerable than girls to the negative consequences of exposure to maternal obesity, as manifested by hippocampal development. There are also sex-dependent differences in susceptibility to neurodevelopmental disorders.

Exposure to maternal obesity also induces alterations in the HPA axis of offspring. Prenatal maternal overnutrition exposes offspring to maternal metabolism abnormalities (e.g. high levels of glucose, TG, and total cholesterol), which are associated with increased activity of the HPA axis, for example, in the means of changes in the children’s cortisol reactivity (262, 263). This is another possible mechanism through which maternal overnutrition may increase the risk of cognitive decline and anxiety-related disorders in offspring (265).

The authors of studies also suggest that there might be a correlation between maternal obesity and increased symptoms of ADHD (266) and autism spectrum disorder in children (267). The analysis of long-term human studies also showed a correlation between maternal obesity and the increased risk of offspring developing anorexia and/or bulimia later in life (268, 269).

## Effects of Maternal Undernutrition on Brain and Behavioural Outcomes in Offspring: Animal Studies

Global caloric and/or protein restriction during gestation is correlated with deficits in exploration, social behaviour, emotionality, avoidance conditioning, learning, and memory in adult life (270–275). Batista et al. observed that maternal protein malnutrition caused autism spectrum disorder in rat offspring (276). Changes in the behaviour of the offspring of malnourished mothers were accompanied by neurochemical and neuroanatomical abnormalities such as: abnormal proliferation, apoptosis, astrogenesis, neuronal differentiation, dendritic arborisation and cerebral astrogliosis in the cortex and hippocampus (277, 278), and lower thickness of the visual cortex, parietal neocortex, dentate gyrus, CA3 region of the hippocampus and cerebellum (276, 279, 280). Experiments on rats also showed that FR during gestation reduced the essential factors enhancing neuronal proliferation, growth, and maintenance, e.g. BDNF and insulin-like growth factor (IGF) (252, 275). Additionally, the offspring of malnourished rats exhibited changes in their central neurochemical profiles, such as altered NMDA transmission and receptor composition, a decrease in the synaptic NO in the hippocampus (252, 281), enhanced GABA-ergic inputs to the hippocampus, and unfavourable changes in the noradrenergic, serotonergic, and dopaminergic transmission (252, 271, 282, 283). Consequently, nutrition-induced deficits in the synaptic transmission impaired memory formation and the LTP (long-term potentiation) (252). Barra et al. suggested that prenatal malnutrition-induced brain disturbances depend on two mechanisms: i) the direct effect of foetal programming on the brain, including negative epigenetic changes in foetal progenitor cells and developing neurons, which will form the cerebral cortex, hippocampus, and other brain regions involved in neuroplasticity, and ii) the indirect effect on the brain mediated by the postnatal development of obesity/metabolic syndrome (271).

Rodriguez et al. conducted research on baboons, which have a similar genetic constitution and developmental trajectory to humans. The researchers observed that moderate global nutrient restriction during pregnancy and lactation affected the development of the offspring brain in a sex-specific manner (45). The baboon offspring exposed to moderate maternal nutrient restriction were less motivated and their working memory was impaired. The female offspring learnt better than the males, whose learning was impaired and they were more impulsive (45).

Natt et al. have described the enhancement of anxiety and depressive behaviour in male, but not female mice offspring from PR mothers (46). However, Akitake et al. have described a decrease in motor activity and recognition memory in male and female mice, while hippocampal synaptophysin level was decreased only in females (10). Additionally, Bellinger et al. in their study on rat offspring of LP mothers, have found that locomotor activity was impaired in males but feeding behaviour was impaired in females (14).

## Effects of Maternal Undernutrition on Brain and Behavioural Outcomes in Offspring: Human Studies

Two meta-analyses described the effects of maternal malnutrition on the learning, memory, and behaviour of the schoolchildren who were small for their gestational age. These children exhibited cognitive impairments, lower verbal and performance IQ, and more behavioural disorders than the children in the control group (271, 284, 285).

Researchers also observed that prenatal exposure to famine (during the Dutch famine) permanently affected the size of the brain, and these changes were sex-dependent. A structural magnetic resonance imaging was conducted on the Dutch famine birth cohort members when they were about 67 years old (54). The MRI showed that the total brain volume as well as the volumes of grey and white matter were smaller only in the early exposed males. The intracranial and total brain volumes of the prenatally exposed males were smaller than the volumes of the control subjects. Such changes were not observed in the females (54). Smith and Reyes described that sons of malnourished mothers are more likely to develop ASD and ADHD than daughters (55).

To sum up, the results discussed above showed that multiple animal models of over- and undernutrition as well as the data obtained in human studies provide convincing insight supporting the influence of an unhealthy maternal diet on the development of the brain and behavioural outcomes in offspring. The authors of these studies also suggested sex-specific responses to maternal diets and proposed various mechanisms underlying such divergences, among which the epigenetic ones have been the most explored recently. Imaging techniques, such as MRI, are invaluable for further analysis of subtle differences in the brain structures of the offspring to obese mothers, with emphasis on possible sex-specific differences.

## Conclusions and Future Directions – From Laboratory to Clinical Settings

This review provided the evidence based on epidemiological data and animal models that both maternal over- and undernutrition changes the metabolic profiles, alters the body weight and fat content, and influences the offspring’s brain development and behaviour. Moreover, the effects observed in offspring are long-lasting. There is a relatively wide range of scientific publications discussing changes in the peripheral nervous system (i.e. hormones and metabolites), but there is not much data on changes in the central nervous system (i.e. in the brain). Non-invasive imaging techniques such as MRI could help to further and more precisely investigate these changes not only within different brain structures but also in the peripheral organs (e.g. changes in the fat distribution). These techniques could be used to monitor the development of diseases and during treatments.

As results from the studies conducted on animals and humans, there are sex-specific differences in the effects of the maternal diet on numerous physiological and anatomical traits. Therefore, further research should be conducted, especially on the epigenetic mechanisms through which both maternal over- and undernutrition affects offspring. These epigenetic processes, including DNA methylation, histone modification, chromatin remodelling, and RNA-based mechanisms may affect gene expression and change the foetal neuroendocrine system through modification of the brain circuitry (286). Epigenetic testing could be used in clinical settings as a biomarker for early diagnosis of the risk of obesity and as a predictor of response to obesity interventions. However, in epigenetic studies on humans it is a challenge to select relevant tissues accessible to examination such as peripheral blood cells (PBC), buccal epithelial cells (BEC), or hair follicles. As both maternal and paternal history and experiences exert influence through the epigenomic information which is not contained in the DNA sequence, including variations in sperm and oocyte cytosine methylation and chromatin patterning, the influence of non-coding RNAs and mitochondria of both maternal and paternal lines should be considered (287, 288). Currently, very little is known about the effects of the paternal metabolic status on the outcomes of offspring. Moreover, the mechanisms involved in the intergenerational transmission of undernutrition, obesity, and diabetes are difficult to study in humans because of the complex relationships between the maternal and foetal conditions, and the postnatal environmental factors.

Therefore, it is also necessary to continue using relevant animal models of metabolic diseases. As the consumption of SSBs is a high risk factor for the development of T2DM, and due to the fact that endocrine disruptors lead to the development of metabolic diseases, animal models with these variables should be developed and employed. Although animal models will never be able to fully mimic the human situation, they make it possible to study the mechanisms through which the mother’s diet programmes the organs and systems of the offspring. Moreover, thanks to animal models of obesity, diabetes, and undernutrition it is possible to search for prevention and treatment strategies, and to increase the awareness of sex-specific risk factors. It is necessary to conduct further research on the sex-dependent pathophysiological mechanisms of undernutrition, obesity, and T2DM, because it could contribute to more personalised care in the future.

The subject of this review seems to be particularly relevant at the time of the pandemic and the post-COVID period, when lockdowns reduce people’s physical activity and increase the consumption of unhealthy food products, which results in higher incidence of obesity. Therefore, it is necessary to develop and implement local, national, and international prevention and treatment strategies to slow down the rate of incidence of obesity. These insights compel us to revise generally held notions to accommodate the prospect that biological parenting, including proper diet, commences well before birth, even prior to conception. As the womb may be more important than home, it is also necessary to urgently implement these strategies long before conception as well as during the crucial and vulnerable times of pregnancy and lactation.

## Author Contributions

EG wrote the draft of the manuscript and especially the following sections: Animal models of obesity with special emphasis on the diet-induced obesity, Effects of maternal overnutrition on body weight and fat content: animal and human studies, Effects of maternal undernutrition on body weight and fat content: human studies, Effects of maternal overnutrition on metabolic and hormonal status of offspring. animal studies, Effects of maternal overnutrition and undermatron on the cardiovascular system in offspring: animal and human studies, Effects of maternal overnutrition and undernutrition on the brain and behavioral outcomes in offspring: animal and human studies. Prepared the figure. JM–collected and formatted literature database. KZ wrote the following sections: Animal models of undernutrition, Effects of maternal undernutrition on body weight and fat content, and metabolic and hormonal status of offspring: animal studies, edited the whole text. AG-K, IK- P, and BS wrote the following sections: Effects of maternal overnutrition on metabolic and hormonal status of offspring: human studies, Effects of maternal undernutrition on metabolic and hormonal status of offspring: human studies and contributed to the Conclusion section; JHS-wrote the following sections: Introduction, Animal models of diabetes, conclusions, overviewed the process of writing the paper. All authors contributed to the article and approved the submitted version.

## Funding

The authors acknowledge the National Science Centre PRELUDIUM grant No. 2018/31/N/NZ4/00518 awarded to KZ, grant No. 2019/35/N/NZ9/00663 awarded to JM and the statutory funding (No. 506-511-09-00) from the Faculty of Veterinary Medicine and Animal Science, Poznań University of Life Sciences, Poland.

## Conflict of Interest

The authors declare that the research was conducted in the absence of any commercial or financial relationships that could be construed as a potential conflict of interest.

## Publisher’s Note

All claims expressed in this article are solely those of the authors and do not necessarily represent those of their affiliated organizations, or those of the publisher, the editors and the reviewers. Any product that may be evaluated in this article, or claim that may be made by its manufacturer, is not guaranteed or endorsed by the publisher.
